# Evaluation of a Clinically Relevant Drug–Drug Interaction Between Rosuvastatin and Clopidogrel and the Risk of Hepatotoxicity

**DOI:** 10.3389/fphar.2021.715577

**Published:** 2021-09-27

**Authors:** Chen Ning, Shengdi Su, Jiaming Li, Dexuan Kong, Hui Cai, Zhiying Qin, Han Xing, Xijing Chen, Jiake He

**Affiliations:** ^1^ Department of Pharmacy, The Second Affiliated Hospital of Nanchang University, Nanchang, China; ^2^ Clinical Pharmacokinetics Laboratory, School of Basic Medicine and Clinical Pharmacy, China Pharmaceutical University, Nanjing, China; ^3^ Department of Cardiovascular Medicine, The Second Affiliated Hospital of Nanchang University, Nanchang, China

**Keywords:** rosuvastatin, clopidogrel, drug–drug interaction, transport, hepatotoxicity

## Abstract

**Purpose:** The combination therapy of rosuvastatin (RSV) and the platelet inhibitor clopidogrel (CP) is widely accepted in the management of cardiovascular diseases. The objective of the present study was to identify the mechanism of RSV–CP DDI and evaluate the risk of hepatotoxicity associated with the concomitant use of CP.

**Methods:** We first studied the effect of CP and its major circulating metabolite, carboxylic acid metabolite (CPC), on RSV transport by overexpressing cells and membrane vesicles. Second, we investigated whether a rat model could replicate this DDI and then be used to conduct mechanistic studies and assess the risk of hepatotoxicity. Then, cytotoxicity assay in hepatocytes, biochemical examination, and histopathology were performed to measure the magnitude of liver injury in the presence and absence of DDI.

**Results:** CP inhibited OATP1B1-mediated transport of RSV with an IC_50_ value of 27.39 μM. CP and CPC inhibited BCRP-mediated RSV transport with IC_50_ values of <0.001 and 5.96 μM, respectively. The CP cocktail (0.001 μM CP plus 2 μM CPC) significantly inhibited BCRP-mediated transport of RSV by 26.28%. Multiple p.o. doses of CP significantly increased intravenous RSV plasma AUC_0-infinity_ by 76.29% and decreased intravenous RSV CL by 42.62%. Similarly, multiple p.o. doses of CP significantly increased p.o. RSV plasma AUC_0-infinity_ by 87.48% and decreased p.o. RSV CL by 43.27%. CP had no effect on cell viability, while RSV exhibited dose-dependent cytotoxicity after 96 h incubation. Co-incubation of 100 μM CP and RSV for 96 h significantly increased intracellular concentrations and cell-to-medium concentration ratios of RSV and reduced hepatocyte viability. Histological evaluation of liver specimens showed patterns of drug-induced liver injury. Cholestasis was found in rats in the presence of DDI.

**Conclusion:** CP is not a clinically relevant inhibitor for OATP1B1 and OATP1B3. The primary mechanism of RSV–CP DDI can be attributed to the inhibition of intestinal BCRP by CP combined with the inhibition of hepatic BCRP by CPC. The latter is likely to be more clinically relevant and be a contributing factor for increased hepatotoxicity in the presence of DDI.

## Introduction

Polypharmacy is a key feature of drug use in clinics that can lead to potential drug–drug interactions (DDIs). Exploring the impact of concomitant drug therapy on drug exposure, efficacy, and safety and, therefore, the ways this might guide drug and dose selection have attracted considerable attention. The antiplatelet agent clopidogrel (CP) is commonly prescribed in the treatment of acute coronary syndrome, myocardial infarction, stroke, and established peripheral arterial disease (Banerjee et al., 2012). At least 85–90% of the absorbed CP is converted into inactive carboxylic acid metabolites (CPC) by carboxylesterase-1 (CES1) in the liver. About 2% of CP undergoes two-step oxidization mediated by cytochrome P450 (CYP) enzymes, forming an active metabolite which possesses antiplatelet aggregation activity (Jiang et al., 2015). CP is excreted as inactive metabolites via urine (∼50%) and feces (∼46%) in 5 days after p.o. administration. The elimination half-lives of CP and CPC are 6 and 8 h, respectively (Sanofi-Aventis, 2011). An epidemiologic study found a strong association between CP use and cerivastatin-induced rhabdomyolysis (Tamraz et al., 2013). The mechanism that is implicated is the inhibition of CYP2C8- and CYP3A4-mediated metabolism of cerivastatin by CP or its metabolites (Floyd et al., 2012). Additionally, CP and its metabolites have been reported to be inhibitors of CYP2B6, CYP2C9, CYP2C19, OATP1B1, and breast cancer resistance protein (BCRP) *in vitro* (Wang et al., 2015; Elsby et al., 2016).

RSV, a hydrophilic statin, is widely used to manage elevated lipid levels and reduce the risk of cardiovascular events in patients with dyslipidemia. While generally safe and effective, the use of RSV is complicated by infrequent but life-threatening side effects in real clinical settings. Cases ranging from liver enzyme abnormalities to hepatic toxicity and from myopathy to rhabdomyolysis have been reported. The incidence of these side effects is dose-dependent and increases in the presence of DDI (Kostapanos et al., 2010). RSV is a multitransporter substrate. The intestinal efflux transporter BCRP plays a vital role in its absorption (Kim et al., 2019). OATP1B1, OATP1B3, and sodium taurocholate cotransporting polypeptide (NTCP) are predominant transporters responsible for the hepatic uptake of RSV with individual contributions of 0.38, 0.11, and 0.21, respectively (Elsby et al., 2016). Canalicular efflux transporters, including multidrug resistance–associated protein 2 (MRP2) and BCRP, participate in the biliary excretion of RSV. Renal organic anion transporter 3 (OAT3) is involved in the renal excretion of RSV (Kim et al., 2019). The metabolism of RSV is minimal. It is eliminated mainly in urine and feces via biliary excretion without any changes (Martin et al., 2003; Jemnitz et al., 2010).

Statins are OATP1B1 substrates (Chauvin et al., 2013). However, DDIs between CP and statins are not consistent. We were surprised to find out that the concomitant use of CP increased the plasma concentration of 40 mg RSV (1.7-fold for AUC and 1.3-fold for C_max_) and 20 mg RSV (2.0-fold for AUC and 2.2-fold for C_max_) but did not change that of simvastatin, fluvastatin, and pitavastatin (Ayalasomayajula et al., 2007; Pinheiro et al., 2012; Remsberg et al., 2013; Itkonen et al., 2015). Besides, there have been reports of liver toxicity during concomitant therapy of RSV and CP (Famularo et al., 2007; Hwang et al., 2015; He et al., 2019). Liver is the site of both action and disposition of RSV and the site of bioactivation of CP. As concomitant use of both medications is common in clinics, it is important to identify the mechanism of RSV–CP DDI and evaluate the risk of hepatotoxicity. Therefore, it was hypothesized that at clinically relevant plasma concentrations, CP or CPC, modulated RSV exposure by the inhibition of uptake (OATP1B1 and/or OATP1B3) and/or efflux transporters (BCRP and/or MRP2) expressed in the liver and/or the intestine, which may contribute to the hepatic events.

To test this hypothesis, the study first determined the effect of CP and CPC on the *in vitro* RSV transport by overexpressing cells (OATP1B1 and OATP1B3) and membrane vesicles (BCRP and MRP2). Second, the study investigated whether a preclinical animal model, such as the rat, could replicate this DDI and then be used to conduct mechanistic studies and assess the risk of hepatotoxicity. Then, cytotoxicity assay in hepatocytes, biochemical examination, and histopathology were performed to measure the magnitude of liver injury in the presence and absence of DDI.

## Materials and Methods

### Chemicals and Reagents

RSV calcium (purity >95%) was purchased from Tokyo Chemical Industry Co. Ltd. (Shanghai, China). CP sulfate (purity >98%) and CPC hydrochloride (purity >98%) were from J&K Scientific Ltd. (Shanghai, China). Propranolol was purchased from National Institutes for Food and Drug Control (Beijing, China). Benzbromarone, tolbutamide, novobiocin, rifampicin, G418 disulfate salt, and reduced glutathione were from Sigma-Aldrich (Shanghai, China). DMEM (low glucose and pyruvate), fetal bovine serum (qualified, Australian origin), pancreatin, and penicillin–streptomycin were obtained from Gibco (Grand Island, NY, United States). The BCA protein assay kit was purchased from Thermo Fisher Scientific Inc. (Shanghai, China) and collagen I-coated multiwell plates were purchased from Corning Inc. (NY, United States). Human OATP1B1 overexpressing HEK293 cells (Cat. No. GM1102G), human OATP1B3 over-expressing HEK293 cells (Cat. No. GM1106G), empty vector-transfected HEK293 cells (mock, Cat. No. GM1001G), human BCRP vesicles (Cat. No. GM0008), human MRP2 vesicles (Cat. No. GM0001), and the vesicular transport assay reagent kit (Cat. No. GM3010) including buffer A2 (reaction buffer; mixture of 50 mM MOPS-Tris, 70 mM KCl and 7.5 mM MgCl_2_), buffer B2 (stopping and washing buffer; mixture of 400 mM MOPS-Tris and 700 mM KCl), reagent C2 (10 mM MgATP solution), and reagent D2 (10 mM MgAMP solution) were provided by GenoMembrane Co., Ltd. (Yokohama, Japan). Cryopreserved human hepatocytes (Lot: DUX) were purchased from BioIVT, LLC (Baltimore, MD, United States). The alanine aminotransferase (ALT) assay kit, aspartate aminotransferase assay (AST) kit, lactate dehydrogenase (LDH) assay kit, total bilirubin (TBIL) kit, and conjugated bilirubin (DBIL) assay kit were provided by Nanjing Jiancheng Bioengineering Institute (Nanjing, China).Analytical grade dimethyl sulfoxide (DMSO) was purchased from Sinopharm Chemical Reagent Co., Ltd. (Beijing, China). Analytical grade ammonium acetate was obtained from Xilong Scientific Co., Ltd. (Shantou, China). HPLC-grade acetonitrile was supplied by Tedia (Fairfield, OH, United States). HPLC-grade formic acid was obtained from Shanghai Aladdin Biochemical Technology Co., Ltd. (Shanghai, China). Ultrapure water used for LC-MS/MS was purified by using a Milli-Q water purification system (Millipore, Bedford, MA, United States). All other chemicals and solvents were of analytical grade and were purchased from Nanjing Chemical Reagent Co., Ltd. (Nanjing, China).

### Animals

A total of 64 male Sprague-Dawley rats (∼9 weeks, 250–300 g) were used in this study (Jiangsu Huayi Technology Co., Ltd., Changshu, China). The rats were housed in a 12 h light-dark cycle, constant temperature (25 ± 2°C), and constant humidity (40–60%) environment with free access to food and water. They were randomly assigned to respective experiment groups. Whenever overnight fasting was used before dosing, food was provided 2 h after dosing. All *in vivo* experiments were carried out in accordance with the Guidelines of the National Institute of Health and were approved by the Institutional Animal Care and Use Committee of the Second Affiliated Hospital of Nanchang University (NO. [2016]097).

### *In Vitro* BCRP and MRP2 Inhibition Assessment of CP and CPC

The vesicular transport activity was determined by the 96-well rapid filtration method in BCRP- and MRP2-expressing membrane vesicles. For samples in the presence of ATP, 10 μL vesicle (final concentration, 1 mg protein/mL), 5 μL buffer A2 (replaced by 100 μM reduced glutathione for MRP2 incubations), 10 μL of varying concentrations of CP or CPC or novobiocin (BCRP inhibitor, final concentration of 100 μM), or benzbromarone (MRP2 inhibitor, final concentration of 100 μM) were pre-incubated at 37°C for 5 min. Meanwhile, 5 μL RSV (final concentration, 1 μM) and 20 μL reagent C2 were pre-incubated at 37°C for 5 min. Then, the transport was initiated by mixing them in a 50 μL reaction system and incubated for the designated time at 37°C. The transport was terminated by adding 200 μL of cold buffer B2 at 4°C. Then, the mixture was filtered using a 96-well filter plate. After washing the vesicles five times with 200 μL of cold buffer B2 at 4°C, the vesicles were lysed with 50 μL of 80% methane twice and then centrifuged at 2000 rpm/min for 2 min. Lysates were collected and then centrifuged at 12,000 rpm/min for 5 min. The supernatants were injected onto the LC-MS/MS. The same procedures were applied to AMP-containing samples except that reagent D2 was used instead of reagent C2. All experiments were performed in quadruplicate.

### *In Vitro* OATO1B1 and OATP1B3 Inhibition Assessment of CP and CPC

The OATP1B1, OATP1B3, and mock cells were cultured in the DMEM medium containing 10% fetal bovine serum, penicillin–streptomycin solution (100 U/mL and 0.1 mg/ml), and 0.5 mg/ml geneticin at 37°C in the presence of 5% CO_2_. The cells were seeded in a 24-well plate at a density of 4×10^5^ cells/well and grown for 80–90% confluence. For the transport study, the cells were washed twice (0.5 ml/each) and pre-incubated with varying concentrations of CP or CPC or rifampin (OATPs inhibitor, final concentration 100 μM) for 30 min at 37°C. Then, the uptake was initiated by replacing the transporter buffer with that of the buffer containing 1 μM RSV with/without varying concentrations of CP or CPC or rifampin (100 μM) and then incubated for 2.5 min at 37°C. After washing the cells three times with transporter buffer at 4°C, the cells were lysed with liquid nitrogen. The lysates were precipitated with methanol and centrifuged at 12,000 rpm at 4°C for 5 min. The supernatant was analyzed by LC-MS/MS. Protein concentration was determined using the Pierce^®^ BCA Protein Assay Kit. All experiments were performed in triplicate.

### Cytotoxicity Assays

Cytotoxicity was determined with MTT assay in cryopreserved human hepatocytes. Thawing and plating of cryopreserved hepatocytes were carried out according to the manufacturer’s protocol. Primary human hepatocytes at a density of 7×10^4^ cells/well were cultured for 24 h after plating in 96-well collagen-coated plates. Then, the hepatocytes were treated with varying concentrations of RSV, CP, RSV plus CP, or tamoxifen and incubated for 24 or 96 h at 37°C. 11 μL of 5 mg/ml MTT was added to each well of the plate, and then the plate was incubated at 37°C for 3–4 h. After the medium was aspirated, 150 μL of DMSO was added to dissolve the formazan. The absorbance at 570 nm was measured and corrected for background absorption at 690 nm using a SpectraMax^®^ i3 plate reader (Molecular Devices, Silicon Valley, CA, United States). AST and LDH levels in the medium of parallel processed plates were measured by commercially available assay kits. The amount of CP, CPC, and RSV in the medium and cell lysate was determined by LC-MS/MS. All experiments were performed in triplicate.

### *In Vivo* Pharmacokinetics Studies

For the plasma pharmacokinetic study, 16 rats were randomly assigned into four groups (n = 4, each group, groups I to IV) and received oral (p.o.) administrations as oral gavage of CP (27 mg/kg) or the same volume of vehicle control for 20 days. Then, the rats were made to fast overnight before dosing but with free access to water. On day 21, rats in group I were treated with a p.o. dose as oral gavage of CP at 27 mg/kg and a single intravenous (i.v.) dose of RSV at 5 mg/kg. Rats in group II were treated with a p.o. dose as oral gavage of vehicle control and a single i.v. dose of RSV at 5 mg/kg. Rats in group Ⅲ were treated with a p.o. dose as oral gavage of CP at 27 mg/kg and a p.o. dose as oral gavage of RSV at 5 mg/kg. Rats in group Ⅳ were treated with a p.o. dose as oral gavage of vehicle control and a p.o. dose as oral gavage of RSV at 5 mg/kg. Blood samples (0.15 ml) were collected at 0, 1, 2, 5, 15, 30, 60, 120, 180, 360, 480, 600, and 720 min after administration of the i.v. dose of the RSV group and at 0, 5, 30, 60, 120, 180, 360, 480, 600, and 720 min for the p.o. dose as oral gavage of the RSV group. After each sampling, a volume of 0.15 ml physiologic saline solutions was administered to sustain isotonic fluid balance. Plasma samples were harvested by using a centrifuge and then stored at −80°C until analysis by LC-MS/MS.

For the hepatobiliary transport study, 24 rats were randomly assigned into eight groups (n = 3, each group, groups V to XII) and they received a p.o. dose as oral gavage of CP (27 mg/kg) for 20 days. The remaining 24 rats were also randomly assigned into eight groups (n = 3, each group, groups XIII to XX) and received the same volume of vehicle control for 20 days. Then the rats were made to fast overnight before dosing but with free access to water. On day 21, rats were anesthetized and incised along the abdominal midline, and bile duct intubation was performed prior to drug administration. Rats in groups V-XII were treated with a p.o. dose as oral gavage of CP at 27 mg/kg and an i.v. dose of RSV at 5 mg/kg. Rats in groups XIII-XX were treated with a p.o. dose as oral gavage of vehicle control and an i.v. dose of RSV at 5 mg/kg. Rats were euthanized at 0.25, 0.47, 0.67, 1, 1.5, 2, 2.5, and 5 min post dose. Arterial blood, bile, and liver samples were collected. Plasma samples were obtained by using a centrifuge, and all samples were stored at −80°C until analysis by LC-MS/MS.

### Biochemical Examination and Histopathology

After the last sample in the plasma pharmacokinetic study, all rats were deeply anesthetized and exsanguinated by severing the abdominal aorta. Blood and liver samples were harvested. The plasma was used for the assay of alanine aminotransferase (ALT), aspartate aminotransferase (AST), total bilirubin (TBIL), unconjugated bilirubin (IBIL), and conjugated bilirubin (DBIL) by an automatic analyzer (Beckman Coulter, Brea, CA, United States). Liver specimens were fixed in 4% paraformaldehyde for 2–3 days and passed through the routine paraffin embedding procedures. They were serially sectioned and stained with hematoxylin and eosin for histological evaluations.

### Bioanalysis

A Shimadzu LC-20AD liquid chromatography system (Shimadzu, Kyoto, Japan) and an API5000 triple quadrupole tandem mass spectrometer (Applied Biosystems/Sciex, Framingham, MA, United States) coupled with an electrospray ionization (ESI) source was used for the determination of *in vitro* samples. Chromatographic separation was conducted with gradient elution on a Synergi Hydro-RP column (30 × 2.0 mm, 4 μm, Phenomenex, Torrance, CA, United States). RSV, CP, CPC, and tolbutamide (internal standard, IS) were detected in the positive ionization mode by multiple reactions monitoring (MRM). The ion transitions of RSV, CP, CPC, and IS were m/z 482.50→m/z 258.10, m/z 322.10→m/z 212.00, m/z 308.00→m/z 198.00, and m/z 271.10→m/z 155.00, respectively. The mobile phase consisted of methanol (A) and water (B), both containing 0.1% (V/V) formic acid. The flow rate was 0.5 ml/min and the gradient elution was run using the following program: 0.01 min (15% A)-1.0 min (95% A), 1.5 min (95% A)-1.51 min (15% A), 2.5 min (stop).

A Shimadzu LC-30AD liquid chromatography system (Shimadzu, Kyoto, Japan) and an API4000 triple quadruple tandem mass spectrometer (Applied Biosystems/Sciex, Framingham, MA, United States) coupled with an ESI source was used for the LC-MS/MS analysis of *in vivo* samples. The separation was carried out on an Agilent Extend C18 column (1.8 µm, 4.6 mm × 30 mm, Agilent Technologies). The mobile phase consisting of acetonitrile (A) and water (B: 1 mM ammonium acetate and 1% (V/V) formic acid). RSV, CP, CPC, and propranolol (IS) were detected in the positive ionization mode by MRM. The ion transitions of RSV, CP, CPC, and propranolol were m/z 482.20→m/z 258.20, m/z 322.10→m/z 212.10, m/z 308.00→m/z 198.30, and m/z 260.00→m/z 116.00, respectively. The flow rate was 1 ml/min and the gradient elution method was as follows: 0.01 min (10% A)-0.4 min (10% A), 0.8 min (95% A)-2.0 min (95% A), and 2.3 min (10% A)-2.5 min (stop).

### Data Analysis

Phoenix WinNonlin^®^ 7.1 (Pharsight Corporation, Mountain View, CA, United States) was used to calculate the pharmacokinetic parameters by the noncompartmental method. IC_50_ values were obtained by a computer-generated nonlinear least squares fit using GraphPad Prism 8.1 (GraphPad Software, San Diego, CA, United States). Statistical analysis was performed using SPSS ver. 22.0 (IBM, Chicago, IL, United States). The results are presented as mean ± S.D., unless otherwise stated. After testing for the homogeneity of variance, statistical analysis was performed with a one-way ANOVA followed by the LSD post hoc test or unpaired Student’s t-test. A probability (p) of less than 0.05, 0.01, and 0.001 was considered to be statistically significant, highly significant, and extremely significant, respectively.

## Results

### RSV Transport by OATP1B1 and OATP1B3 Overexpressing HEK293 Cells

**FIGURE 1:**
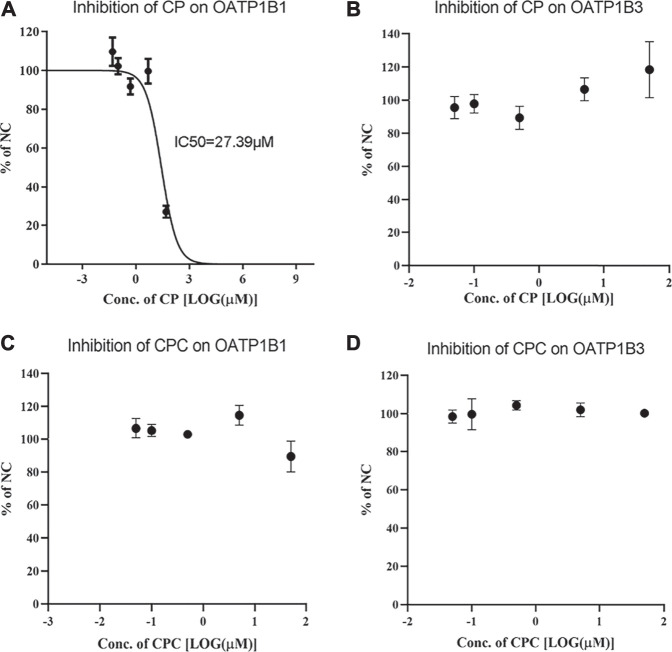
Inhibition potency of CP and CPC on OATP1B1- or OATP1B3-mediated RSV transport. The mean relative uptake activity (% of NC) of RSV by OATP1B1 **(A and C)**, OATP1B3 **(B and D)** with varying concentrations of CP or CPC. Data are presented as the mean ± S.D. (*n* = 3).

The uptake of RSV by OATP1B1 and OATP1B3 overexpressing HEK293 cells was significantly inhibited by 100 μM rifampin, indicating robust transport activity of OATP1B1 and OATP1B3 in these cells. Although CP did not inhibit OATP1B3-mediated RSV transport, it did inhibit OATP1B1-mediated transport of RSV with an IC_50_ value of 27.39 μM (Figures 1A,B). In contrast, CPC did not inhibit RSV transport by OATP1B1 or OATP1B3 in the concentration range of 0.05–50 μM (Figures 1C,D).

### RSV Transport by BCRP- and MRP2-Expressing Membrane Vesicles

**FIGURE 2:**
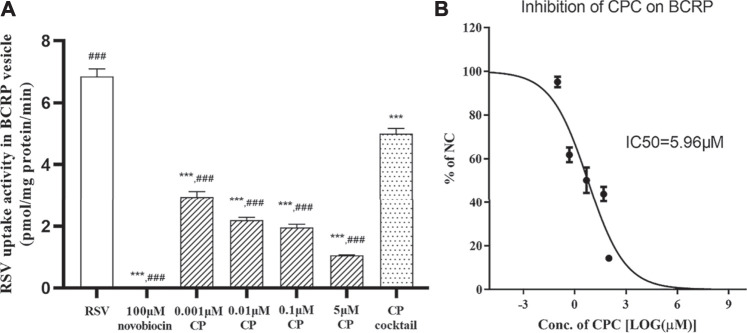
Inhibition of novobiocin, CP, CP cocktail, or CPC on the RSV transport in BCRP-expressing membrane vesicles. The mean uptake activity of RSV by BCRP in the presence and absence of novobiocin, CP cocktail (0.001 μM CP plus 2 μM CPC), and varying concentrations of CP **(A)**. The mean relative uptake activity (% of NC) of RSV by BCRP with varying concentrations of CPC **(B)**. Data are presented as the mean ± S.D. (n = 4). ***p < 0.001 vs RSV. ^###^ *p* < 0.001 vs CP cocktail.

The uptake of RSV into BCRP-expressing membrane vesicles was significantly inhibited by 100 μM novobiocin and in the presence of AMP, which indicated robust transport activity of BCRP in the vesicle test system. CP significantly inhibited the BCRP-mediated transport of RSV with an IC_50_ < 0.001 μM (56.93% inhibition at 0.001 μM; Figure 2A). CPC inhibited the BCRP-mediated transport of RSV with an IC_50_ value of 5.96 μM (Figure 2B). Moreover, at clinically relevant unbound plasma concentration, the CP cocktail (0.001 μM CP plus 2 μM CPC) significantly inhibited the BCRP-mediated transport of RSV by 26.28% (Figure 2A). However, RSV did not exhibit ATP- and time-dependent uptake into MRP2-expressing membrane vesicles for an uptake ratio less than 2 (data not shown).

### Cytotoxicity and Cellular Accumulation

**FIGURE 3:**
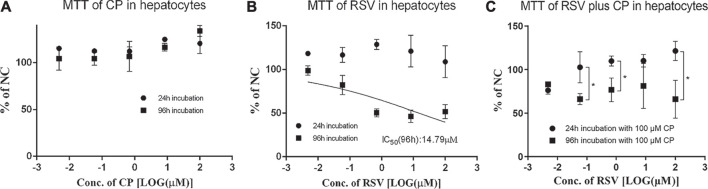
Effects of CP and RSV on the viability of human primary hepatocytes. The mean relative cell viability (% of NC) of the human primary hepatocytes in the presence of CP **(A)**, RSV **(B),** and RSV plus CP **(C)**. Data are presented as the mean ± S.D. (n = 3). *p < 0.05.

**FIGURE 4:**
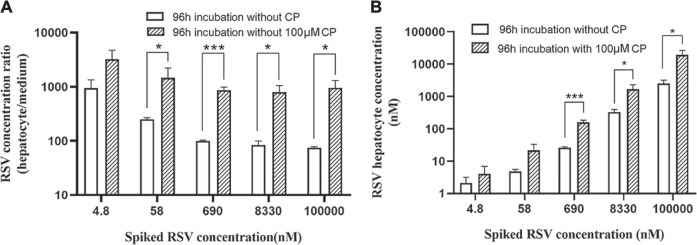
Cell to medium concentration ratio **(A)** and intracellular concentration of RSV **(B)** in the presence and absence of CP after 96 h incubation. Data are presented as the mean ± S.D. (n = 3). *p < 0.05, ***p < 0.001 vs 96 h incubation without CP.

Cytotoxicity of CP, RSV, and CP plus RSV after short-term (24 h) and long-term (96 h) incubation was assessed by the MTT test (Figure 3). Dose-dependent cytotoxicity of tamoxifen (positive control) was observed after both 24 and 96 h incubations (Supplementary Figure S1A). CP did not show hepatotoxic effects during incubation. Dose-dependent cytotoxicity of RSV was only observed after 96 h incubation, with an IC_50_ value of 14.79 µM. Co-incubation of 100 µM CP and RSV for 24 h did not produce significant toxicity in primary hepatocytes, but co-incubation of 100 µM CP and RSV for 96 h significantly affected cell viability. A trend of increase in the AST level was observed in the presence of 100 µM CP, but it missed statistical significance, as did LDH (Supplementary Figure S1B,C). The cell to medium concentration ratios and intracellular concentrations of RSV after 96 h co-exposure were also significantly enhanced (Figures 4A,B). In addition, CPC concentrations in cells and mediums remained high and appeared unaffected in the presence of varying concentrations of RSV, while intracellular and extracellular CP concentrations were extremely low (Supplementary Figure S1D).

### Pharmacokinetics of RSV in the Presence and Absence of CP in Rats

The accuracy (bias, %) or precision (CV, %) in the assay of RSV, CP, and CPC, based on quality control (QC) samples that spanned the calibration range, was <15%. Additional sextuplicate QC samples at concentrations 10 times higher than the upper limits of the calibration were used to test the dilution integrity. The dilution samples with a dilution factor of 20 also met the acceptance criteria of ±15% RSD for accuracy and precision (Supplementary Table S1).

**FIGURE 5:**
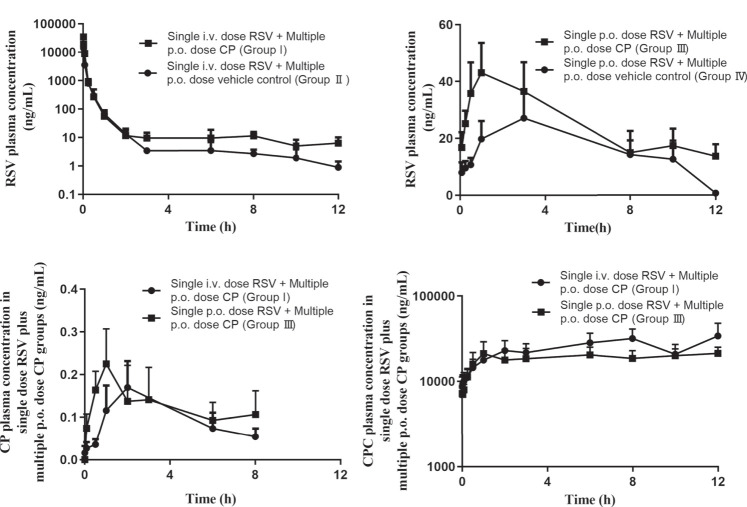
Mean plasma concentration-time profiles of RSV **(A and B)**, CP **(C)**, and CPC **(D)** in rats. Data are presented as the mean ± S.D. (n = 4). p.o. dose: p.o. administration as oral gavage.

The plasma concentration after i.v. administration of RSV decreased biphasically in the presence and absence of CP (Figure 5A). When RSV was given intravenously to rats, multiple p.o. doses of CP significantly decreased RSV CL by 42.62%, increased plasma AUC_0–12h_ and AUC_0-infinity_ by 75.55 and 76.29%, respectively, but did not significantly affect the volume of distribution at steady-state (V_ss_) and T_1/2_ (group I). Although RSV C_max_ was increased by 110.32%, it failed to elicit significant changes (Table 1). RSV bile_0–5min_/liver AUC_0–5min_, CL_int,bile_, liver AUC_0–5min_/plasma AUC_0–5min_, and CL_uptake,liver_ decreased by 26.19%, 39.19%, 19.77%, and 52.38%, respectively, while they missed statistical significance (Supplementary Table S2).

**TABLE 1:** Pharmacokinetic parameters of RSV, CP, and CPC in the presence and absence of CP. Data are presented as mean ± S.D. (n = 4). *p < 0.05 when compared with the respective single dose RSV group. The rats were randomly assigned into four groups (n = 4, each group, group I to IV) and each group received a p.o. dose of CP (27 mg/kg) or the same volume of vehicle control for 20 days. On day 21, rats in group I were treated with a p.o. dose of CP at 27 mg/kg and a single i.v. dose of RSV at 5 mg/kg. Rats in group II were treated with a p.o. dose of vehicle control and a single i.v. dose of RSV at 5 mg/kg. Rats in group Ⅲ were treated with a p.o. dose of CP at 27 mg/kg and a p.o. dose of RSV at 5 mg/kg. Rats in group Ⅳ were treated with a p.o. dose of vehicle control and a p.o. dose of RSV at 5 mg/kg.

Pharmacokinetic parameters		Group I Single i.v. dose RSV + multiple p.o. dose CP	Group II Single i.v. dose RSV + multiple p.o. dose vehicle control	Group Ⅲ Single p.o. dose RSV + multiple p.o. dose CP	Group Ⅳ Single p.o. dose RSV + multiple p.o. dose vehicle control
RSV					
	T_1/2_(h)	1.70 ± 0.34	1.52 ± 0.56	6.98 ± 6.27	5.82 ± 3.73
	T_max_(h)			1.25 ± 1.19	4.25 ± 3.95
	C_max_ (ng/mL)	34,550.00 ± 17,983.23	16,427.50 ± 8,389.02	47.63 ± 19.40	33.10 ± 13.50
	AUC_0–12h_ (ng×h/mL)	2,630.36 ± 874.65*	1,498.37 ± 437.64	326.03 ± 38.24	194.91 ± 113.36
	AUC_0-infinity_ (ng×h/mL)	2,646.98 ± 894.00*	1,501.46 ± 439.38	503.84 ± 185.28*	268.74 ± 67.25
	CL (CL/F) (ml/h/kg)	2035.26 ± 594.04*	3,547.30 ± 999.88	10,970.26 ± 3,887.00*	19,338.35 ± 3,911.35
	V_ss_ (V_ss_/F) (ml/kg)	924.83 ± 406.14	1,148.40 ± 701.23	86,656.05 ± 49,504.43	171,328.87 ± 125,748.05
CP					
	T_1/2_(h)	4.18 ± 1.69			
	T_max_(h)	3.00 ± 2.00		2.88 ± 3.47	
	C_max_ (ng/mL)	0.18 ± 0.12		0.27 ± 0.16	
	AUC_0–12h_ (ng×h/mL)	0.86 ± 0.51		1.02 ± 0.69	
	AUC_0-infinity_ (ng×h/mL)	1.17 ± 0.70			
CPC					
	AUC_0–12h_ (ng×h/mL)	299,764 ± 153,504.1		230,181 ± 109,005.9	

po. dose: p.o. administration as oral gavage.

When RSV was given orally to rats, multiple p.o. doses of CP significantly decreased RSV CL by 43.27% and increased plasma AUC_0–12h_ and AUC_0-infinity_ by 67.27 and 87.48%, respectively, but did not significantly affect T_1/2_ and T_max_ (group Ⅲ). A 43.90% increase in RSV C_max_ and a 49.42% decrease in V_ss_ were also observed, while they failed to elicit significant changes (Table 1).

There were no significant differences in the pharmacokinetic parameters of CP and CPC in groups I and Ⅲ, which suggested that the RSV dosing regimen had no effect on CP metabolism. The plasma concentrations of CP were undetectable after 8 h post-dosing, but the exposure of CPC remains high at 12 h post-dose (Figures 5C,D).

### Biochemical Examination and Histopathology

**FIGURE 6:**
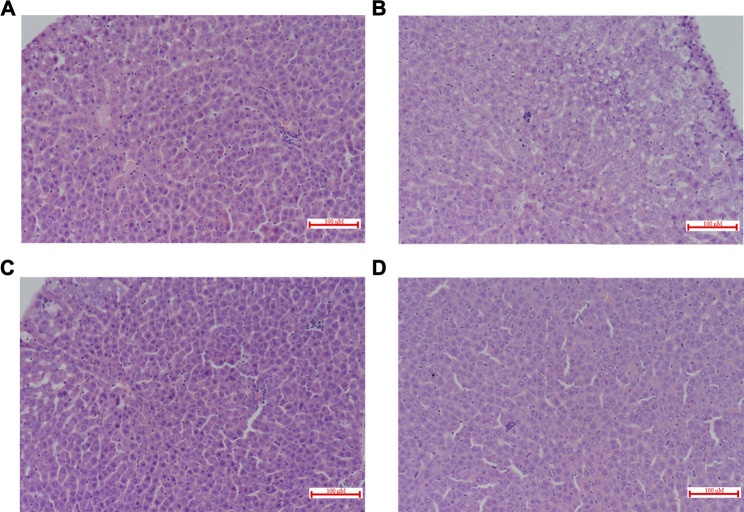
Histopathological evaluation of rat livers 12 h after the administration of RSV in combination with CP (H&E staining, ×200). The liver sections **(A)** Single i.v. dose RSV + Multiple p.o. dose CP (Group I); **(B)** Single i.v. dose RSV + Multiple p.o. dose vehicle control (Group II); **(C)** Single p.o. dose RSV + Multiple p.o. dose CP (Group Ⅲ); **(D)** Single p.o. dose RSV + Multiple p.o. dose vehicle control (Group Ⅳ). p.o. dose: p.o. administration as oral gavage.

There were no significant changes in ALT and AST. However, co-administration of CP and RSV significantly increased (100%, *p* = 0.032) DBIL in group Ⅲ (Table 2). Histological patterns of drug-induced liver injury including steatosis (macrovesicular and/or microvascular), scattered apoptosis and spotty necrosis with lymphocyte aggregation, and scattered eosinophils in the portal area were observed in all liver specimens (Figure 6). Cholestasis was found only in group I and group Ⅲ (Figures 6A,C). Plasma cell infiltration and interfacial hepatitis were also observed in group I and group Ⅲ, which were signs of autoimmune hepatitis.

**TABLE 2:** Liver function indices of rats. Data are mean ± S.D. (n = 4). *p < 0.05 when compared with the respective single dose RSV group.

Liver function index	Group I single i.v. dose RSV + multiple p.o. dose CP	Group II single i.v. dose RSV + multiple p.o. dose vehicle control	Group Ⅲ single p.o. dose RSV + multiple p.o. dose CP	Group Ⅳ single p.o. dose RSV + multiple p.o. dose vehicle control
ALT (U/L)	46.50 ± 11.27	54.00 ± 14.17	54.50 ± 18.43	52.00 ± 6.68
AST (U/L)	169.75 ± 17.93	210.50 ± 36.59	196.00 ± 36.28	212.50 ± 33.72
TBIL (μmol/L)	1.95 ± 0.44	2.00 ± 0.22	2.00 ± 0.49*	1.13 ± 0.42
DBIL (μmol/L)	0.89 ± 0.42	1.12 ± 0.11	1.16 ± 0.17*	0.58 ± 0.33
IBIL (μmol/L)	1.06 ± 0.21	0.88 ± 0.18	0.85 ± 0.33	0.57 ± 0.36

p.o. dose: p.o. administration as oral gavage.

## Discussion

The prevalence of multimorbidity and polypharmacotherapy has posed a challenge for patient safety in clinics, because the risk of DDIs and associated adverse effects are massively increasing. The combination therapy of RSV and CP is widely accepted in the management of cardiovascular diseases. RSV is generally safe, effective, and well-tolerated. It exhibits minimal hepatic metabolism and does not reduce the ability of CP to inhibit platelet aggregation, which makes RSV a preferred option for patients (Benes et al., 2016). However, as a substrate of multiple transporters, RSV is likely to act as a victim of transporter-related DDIs in the presence of polypharmacy. Currently, RSV is the only statin that has clinically relevant DDI with CP in coronary heart disease patients (Serebruany et al., 2004; Pinheiro et al., 2012). Dosage of RSV and DDIs appear to be more important regarding the risk of hepatic events. Therefore, it is necessary to characterize the mechanism of RSV–CP DDI and evaluate the risk of hepatotoxicity associated with the concomitant use of CP.

OATPs-mediated RSV uptake is a rate-limiting step in RSV disposition (Uchida et al., 2018). CP inhibited the OATP1B1-mediated transport of RSV with an IC_50_ value of 27.39 μM, but it did not affect OATP1B3-mediated RSV uptake. The obtained IC_50_ value was several-fold greater than the reported values where cerivastatin or estradiol 17β-glucuronide was used as the substrate (Table 3). This observed discrepancy may be attributed to substrate-dependent inhibition of OATP1B1 (Izumi et al., 2014). CP was not likely to cause a clinically relevant DDI with RSV through inhibition of hepatic OATP1B1 ([I_in, max,u_]/IC_50 OATP1B1_<0.1; Table 3). Additionally, CPC had no effects on OATP1B1- and OATP1B3-mediated RSV transport *in vitro*. Inactive CP acyl-β-glucuronide and 2-oxo-clopidogrel were two other inactive metabolites of CP and were reported to inhibit OATP1B1-mediated cerivastatin uptake with IC_50_ values of 10.9 and 8.18 μM, respectively (Tamraz et al., 2013). However, on account of their low concentrations ([I_max,u_]/IC_50 OATP1B1_ < 0.1; Table 3), they were unlikely to predict clinically relevant DDIs even at a loading dose of 300 mg CP. Collectively, these data indicate that clinical dose of CP likely exerts no effect on OATP1B1- and OATP1B3-mediated hepatic uptake *in vivo*, which is also confirmed by the fact that CP has no clinically significant DDIs with simvastatin, fluvastatin, and pitavastatin (Kim et al., 2016).

**TABLE 3:** Summary of inhibitory properties and pharmacokinetic parameters of clopidogrel (CP), clopidogrel carboxylic acid (CPC), clopidogrel acyl-β-D-glucuronide, 2-oxo clopidogrel at clinically relevant doses.

Perpetrator	MW	f_u_	IC_50__OTAP1B1_	IC_50__OTAP1B3_	IC_50__BCRP_	Dose	I_1_	I_2_	I_max,u_	I_in,max,u_	DDI potential for intestinal efflux transporter	DDI potential for hepatic uptake transporter	DDI potential for canalicular efflux transporter
			μM		μM	mg	μM	μM	μM	μM	Cut-off:10; 0.1	Cut-off: 0.1	Cut-off: 0.1
Clopidogrel	321.822	0.02 Tornio et al. (2014)	3.95(CER) Tamraz et al. (2013)1.8 (e17βG) Elsby et al. (2016)27.39^※^	>50^※^	63 Elsby et al. (2016)104.3 Varma et al. (2019)≈0.001^※^	75	0.0010 Itkonen et al. (2015)	932.19	0.00002	0.31	I_2_/IC_50 BCRP_ >> 10	I_in,max,u_/IC_50 OATP1B1_ = 0.011	I_max,u_/IC_50 BCRP_≈0.02
						300	0.0089 Itkonen et al. (2015)	3,728.77	0.00018	1.24	I_2_/IC_50 BCRP_ >> 10	I_in,max,u_/IC_50__OATP1B1_ = 0.045	I_max,u_/IC_50 BCRP_ ≈ 0.18
Clopidogrel carboxylic acid derivative	307.795	0.06 Tornio et al. (2014)	>100 (CER; ES) Tamraz et al. (2013) > 50^※^	>50^※^	5.96^※^	75	7.38 Itkonen et al. (2015)	N/A	0.443	N/A	I_max,u_/IC_50 BCRP_ = 0.074	I_max,u_/IC_50__OATP1B1_ < 0.1	I_max,u_/IC_50 BCRP_ = 0.074
						300	31.90 Itkonen et al. (2015)	N/A	1.914	N/A	I_max,u_/IC_50 BCRP_ = 0.321	I_max,u_/IC_50__OATP1B1_ < 0.1	I_max,u_/IC_50 BCRP_ = 0.321
Clopidogrel acyl glucuronide	483.919	0.1 Tornio et al. (2014)	10.9 (CER) Tamraz et al. (2013)33.5 (ES) Tamraz et al. (2013)	N/A	N/A	75	1.72 Itkonen et al. (2015)	N/A	0.172	N/A	N/A	I_max,u_/IC_50__OATP1B1_ = 0.016	N/A
						300	4.77 Itkonen et al. (2015)	N/A	0.477	N/A	N/A	I_max,u_/IC_50 OATP1B1_ = 0.044	N/A
2-Oxo clopidogrel	337.821	0.02 Tornio et al. (2014)	8.18(CER) Tamraz et al. (2013)8.97 (ES) Tamraz et al. (2013)	N/A	N/A	75	0.004	N/A	0.00008	N/A	N/A	I_max,u_/IC_50__OATP1B1_ << 0.1	N/A
						300	0.016 Kim et al. (2016)	N/A	0.0003	N/A	N/A	I_max,u_/IC_50__OATP1B1_ << 0.1	N/A

MW, molecular weight; f_u_, fraction unbound in human plasma; I_1_, peak plasma concentrations at steady state of CP, CPC, clopidogrel acyl-β-D-glucuronide, and 2-oxo clopidogrel were obtained from two clinical trials (Itkonen et al., 2015; Kim et al., 2016) except that I_1_ of 2-oxo clopidogrel at a maintenance dose of 75 mg CP was calculated from the I_1_ value at a loading dose of 300 mg CP assuming linear pharmacokinetics; I_2_, maximal theoretical gastrointestinal concentration (calculated from dose [mol]/250 ml); I_max,u_, unbound steady-state plasma peak concentration, calculated as fu×I_1_; I_in,max,u_, steady-state unbound liver inlet concentration, calculated as fu× [I_1_ + (F_a_×k_a_×Dose/Q_h_)], where F_a_ is the fraction absorbed (taken to be 1.0 as the default value), k_a_ is the absorption rate constant (as default taken to be 0.1 min^−1^), Q_h_ is hepatic blood flow (1,500 ml/min) (U.S. Department, 2020); IC_50_, half maximal inhibitory concentration; ^※^IC_50_ value obtained from this study using rosuvastatin (RSV) as a substrate; e17βG, estradiol 17β-glucuronide; CER, cerivastatin; ES, estrone sulfate; and NA, not applicable. Whenever IC_50_ values obtained with RSV as a substrate were unavailable, the smallest value among all reported IC_50_ values obtained with other substrates (e.g., CER) was selected for the worst case estimate. The DDI potentials of different transporters were calculated according to the recommended equations (U.S. Department, 2020; Parkinson, 2019). For the intestinal efflux transporter, DDI potential of the orally administered drug was I_2_/IC_50_, and the cut-off value was 10; for the metabolite of the orally administrated drug, the DDI potential was I_max,u_/IC_50_, and the cut-off value was 0.1; for the hepatic uptake and biliary efflux transporters, DDI potential of both parent forms or the metabolite was I_in,max,u_/IC_50_, and the cut-off value was 0.1. All IC_50_ values used for the calculation of DDI potentials were from this study except the IC_50OTAP1B1_ value of clopidogrel acyl-β-D-glucuronide and 2-oxo clopidogrel, and the DDI potential for OATP1B3 was not calculated because the inhibitory effect was not obtained in this study or from previous reports.

The effects of CP and CPC on MRP2- and BCRP-mediated transport of RSV were examined. However, we were unable to obtain significant ATP-dependent accumulation of RSV in MRP2-expressing membrane vesicles. The involvement of MRP2 in the biliary efflux of RSV remains controversial. Some studies using vesicular transport systems similar to ours reported no detectable MRP2-mediated transport of RSV, but others employing OATP1B1/MRP2 double transfectants, sandwich-cultured rat hepatocytes, Mrp2-deficient rats, or ATPase assay found that MRP2/Mrp2-mediated transport occurred for RSV (Johnson et al., 2005; Huang et al., 2006; Abe et al., 2008; Kitamura et al., 2008; Ellis et al., 2013). This might be due to the different transport efficiencies between human MRP2 and rat Mrp2 or other endogenous MRP2-like proteins expressed in the vesicles. Additionally, the inherent disadvantage of membrane vesicles is that compounds with medium-to-high passive permeability are not retained inside the vesicles, making direct transport measurements with this class of compounds difficult to perform (Glavinas et al., 2008). MRP2 vesicles may not be an ideal model to test the transport of RSV. Considering the lack of clinical reports on the effect of ABCC2 polymorphism on PK/PD of RSV, further studies using triple transfectants or humanized MRP2 rodents are needed to investigate the contribution of MRP2 to the biliary efflux of RSV (Ieiri et al., 2009).

We found that CP and CPC were inhibitors of BCRP-mediated RSV transport with IC_50_ values <0.001 and 5.96 μM, respectively. The maximal theoretical gastrointestinal concentration of CP at 75 and 300 mg were 932.19 and 3,728.77 μM, respectively. CP was predicted to cause a DDI through intestinal BCRP inhibition *in vivo* ([I_2_]/IC50 BCRP ratios >10; Table 3). The unbound fractions of CP and CPC were 0.02 and 0.06, respectively. The I_max,u_ values of CP and CPC at 75 mg were calculated to be 0.02 and 0.18 nM, respectively. Therefore, at a maintenance dose of 75 mg, the [I_max,u_]/IC50 BCRP values for CP and CPC were approximately 0.02 and 0.074, respectively, indicating a remote possibility of DDI (<cutoff value 0.1). However, the [I_max,u_]/IC_50BCRP_ value for CP and CPC increased to 0.18 and 0.321, respectively, indicating a high DDI potential in the presence of 300 mg CP. In real clinical settings, a 300–600 mg loading dose of clopidogrel followed by a 75 mg/d maintenance dose is widely accepted. And a 900-mg clopidogrel loading dose induced a greater antiplatelet effect than the 600 mg regimen (Montalescot et al., 2006). The increased CP dose also increased the risk of RSV-CP DDI based on the I/IC_50_ value calculation. CP is converted into CPC by hepatic CES1. Considering the accumulation of CPC in hepatocytes, the inhibition of hepatic BCRP by CPC may be underestimated. Elsby et al. and Varma et al. evaluated the inhibitory efficacy of CP on BCRP-mediated RSV transport by Caco-2 cells and membrane vesicles, and the reported IC_50_ values were 63 μM and 104.3 μM, respectively (Elsby et al., 2016; Varma et al., 2019). Our observed significant inhibition of BCRP-mediated RSV transport by CP was a surprise. Substantial interlaboratory variabilities in the estimation of IC_50_ values were not rare. Using miscellaneous *in vitro* experimental systems, a P-glycoprotein IC_50_ working group reported an 18- and 796-fold difference between the lowest and highest IC_50_ values, respectively (Bentz et al., 2013). Caco-2 cells are known to express many endogenous transporters in addition to BCRP, which may interfere with the IC_50_ value estimation. The vesicles in Varma’s study were prepared from the BCRP overexpression of HEK cells, while the vesicles in this study were prepared from an insect cell system (Sf9 cells) expressing human BCRP. It is possible that differences in the expression systems, vesicle preparation procedures, as well as differences in inhibition study protocols, contributed to the interlaboratory variabilities. To further explore the effect of CP at clinically relevant unbound concentration, the BCRP-mediated RSV transport was measured using the CP cocktail (0.001 μM CP plus 2 μM CPC). The CP cocktail caused significant BCRP inhibition, but the magnitude was attenuated. BCRP has more than one substrate binding site and displays biphasic kinetics (Mao and Unadkat, 2014). CP and CPC may have different binding affinities to BCRP. The mutual interactions between CP and CPC are likely to induce conformational changes of BCRP and could allosterically affect its function. Although inter-inhibitor interaction is a less understood area and the high-resolution 3D structure of BCRP has been unavailable, the development of ligand-based computational methods and homology models seems to be a feasible way to investigate the complex interaction mechanism (Matsson et al., 2007; Gantner et al., 2017).

Rats are commonly used for pharmacokinetic assessments and toxicity evaluation in drug discovery. To elucidate the mechanisms of RSV-CP interaction, we asked whether a rat model could replicate this DDI and explore the risk of hepatic injury in the presence of DDI. The aim of multiple p.o. doses rather than a single dose of CP was to mimic clinical scenarios and to inhibit or stimulate enzymes or transporters if any. In our rat study, the pharmacokinetic parameters of RSV in control groups were consistent with previously reported values (Nezasa et al., 2002). Considerable decreases in RSV hepatic uptake and excretion were observed in the presence of CP, but they missed statistical significance due to large variability (Supplementary Table S2). Therefore, further studies using PET imaging are needed to determine the contribution of various transporters to the hepatobiliary clearance and hepatic exposure of RSV in the presence of DDI. The presence of CP significantly increased i.v. and p.o. RSV AUC to 1.76-fold and 1.87-fold, respectively, which was comparable to that in human reports (1.7-fold at 40 mg RSV and 2.0-fold at 20 mg RSV) (Pinheiro et al., 2012; Remsberg et al., 2013). And CP significantly reduced RSV CL by 42.6% and 43.3%, respectively. The probable reason is the inhibition of both intestinal and hepatic BCRP in the presence of CP and CPC. The systemic disposition of CP and CPC was not affected by RSV (Lee et al., 2012; Chen et al., 2015). By assuming the same unbound fraction of CP (0.02) and CPC (0.06) in rat and human plasma, only the unbound concentrations of CPC in group I and group Ⅲ falls in the range of CPC-mediated BCRP inhibition, which are estimated to be 3.74 and 5.05 μM, respectively (Tornio et al., 2014). Considering the high sequence homology of BCRP/Bcrp (81–86%) between rodents and humans, we expected minimal species differences in substrate specificity (Doyle and Ross, 2003).

Liver injury associated with statins is rare but is associated with severe outcomes. The clinical use of RSV has been associated with an increase in transaminase levels, jaundice, and autoimmune hepatitis (Kasliwal et al., 2007). In order to mimic the clinical scenario of chronic administration, a 96-h incubation period was selected. CP had no effect on human hepatocyte viability by the MTT assay. RSV only exhibited dose-dependent cytotoxicity after long-term incubation for 96 h. A previous study also reported significant dose-dependent toxicity of RSV for 72 h in mouse primary hepatocytes (Kah et al., 2012). Similarly, significantly reduced hepatocyte viability was observed after 96 h co-incubation of RSV and 100 μM CP. Hepatotoxicity is considered to be dependent on the amount of RSV in the hepatocytes. Thus, the intracellular concentration and cell-to-medium (C/M) concentration ratios of RSV were further assessed with the inclusion of compromised cell viabilities. We found a long-term co-exposure of RSV and 100 μM CP significantly increased the intracellular concentration and C/M concentration ratios of RSV. Assuming the same unbound fraction in hepatocytes, medium, and plasma, the free medium concentrations of CP and CPC are estimated to be 0.026–0.40 nM and 0.79–1.31 μM, respectively, which barely inhibit OATP1B1-mediated RSV uptake on their own. And the unbound intracellular concentrations of CPC are estimated to be 7.42–9.11 μM, which is capable to inhibit BCRP-mediated RSV efflux. Put together, these data suggest that the intracellular accumulation of RSV through BCRP inhibition is responsible for increased *in vitro* cytotoxicity.

Primary human hepatocytes remain the gold standard for *in vitro* testing of drug metabolism and cytotoxicity (LeCluyse, 2001). However, the disadvantage of this system is that the absolute abundance and/or the activity of the transporter protein may be decreased when the hepatocytes are cultured. Recent studies have reported that the protein expression of sinusoidal OATPs/Oatps in both sandwich-cultured hepatocytes and cryopreserved hepatocytes is lower than that in the liver tissue (Kimoto et al., 2012; Ishida et al., 2018). Large interindividual differences in OATPs levels were also reported (Prasad et al., 2014). This lower expression has resulted in inappropriate scaling factors and underprediction of the *in vivo* hepatic uptake clearance. RSV was the substrate of OATP1B1 and OATP1B3. The reduced expression of functional sinusoidal uptake transporters in the conventional culture of human hepatocytes might make this system not very relevant when extrapolating hepatotoxicity from *in vitro* to *in vivo*. Thus, cryopreserved hepatocytes should be used with awareness of their limitations. Sandwich-cultured hepatocytes with quantitative proteomics analyzed by mass spectrometry would be considered a superior model.

The histological evaluation of liver specimens showed patterns of drug-induced liver injury (DILI) in all groups, which were consistent with the clinical observations (Russo et al., 2009). It is interesting to note that cholestasis was found only in rats after co-administration of CP and RSV (group I and group Ⅲ), indicating impaired biliary excretion of RSV. In addition, multiple p.o. doses of CP significantly increased plasma conjugated bilirubin (DBIL) level by 2-fold in group Ⅲ. BCRP/Bcrp has been identified to play an important role in conjugated bilirubin excretion (Memon et al., 2016). It is possible that the inhibition of BCRP/Bcrp-mediated RSV efflux by CPC contributes to cholestasis. Due to differences in transporter expression and catalytic activity, the quantitative contribution of the various transporters to the disposition and local tissue toxicity of RSV in humans will differ from that in the rat. The direct data extrapolation from rats to humans may be misleading, and the interpretation of rat data should be performed with caution.

Nowadays, concomitant use of CP and RSV is widely accepted in clinical practices, especially in patients with acute coronary syndromes (ACS) and those who underwent percutaneous coronary intervention (PCI) for the prevention of stent thrombosis. Moreover, in the management of ACS, lipid-lowering treatment with high-intensity statin therapy, such as rosuvastatin, is advised for secondary prevention of cardiovascular events over the long term (Virani et al., 2020). Once-daily CP can be administered either in the morning or in the evening in clinical practice, while RSV is recommended to be taken in the evening. When staggered dosing of CP and RSV is adopted, the intestinal CP concentration (I_2_) should be much lower than dose/250 ml at the time of RSV dosing. This dosing strategy is able to attenuate the magnitude of RSV–CP DDI and could be a better way to minimize the associated side effects.

**FIGURE 7:**
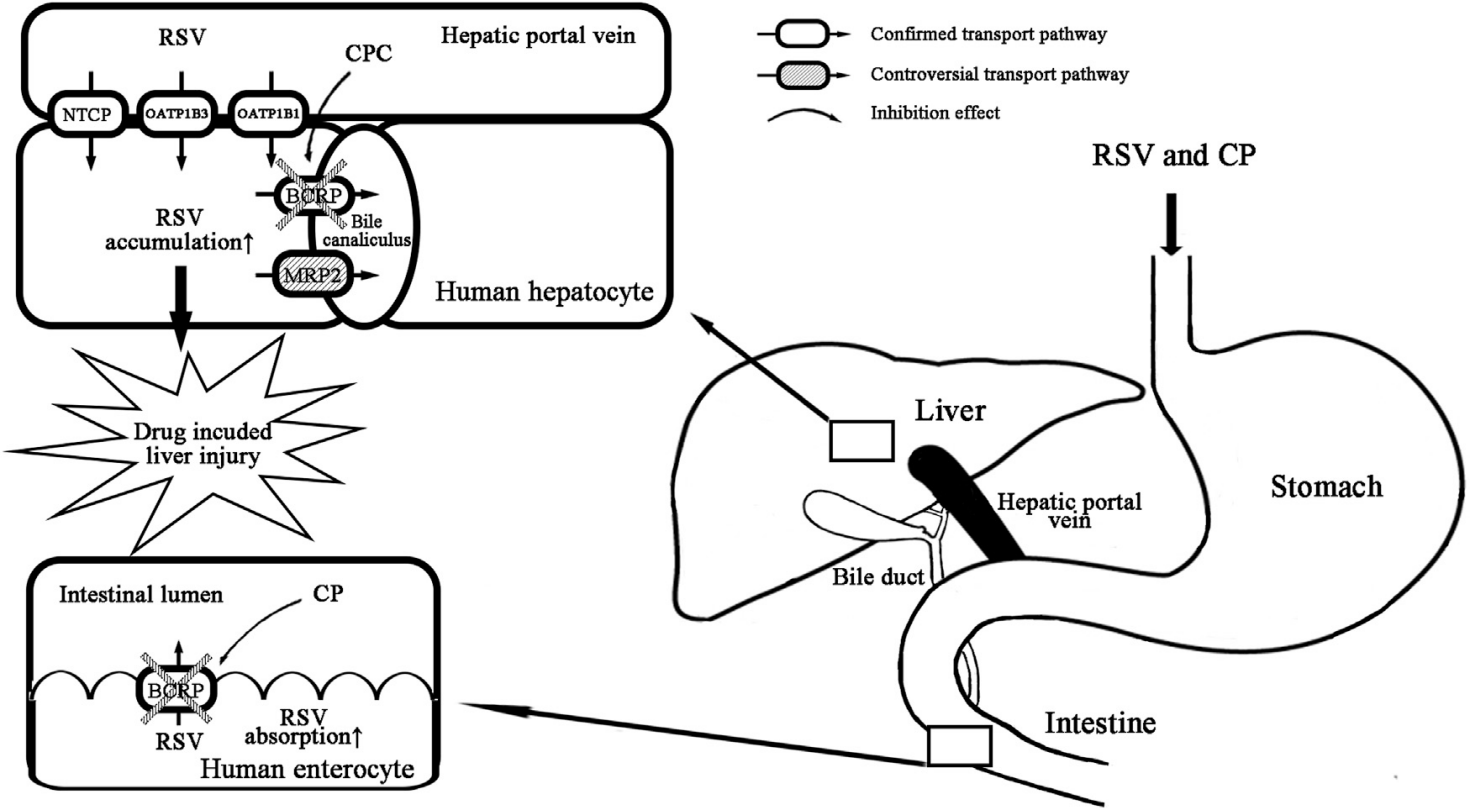
Proposed mechanism of RSV-CP DDI. CP inhibits intestinal BCRP efflux, and CPC inhibits hepatic BCRP efflux of RSV.

In conclusion, we clarify that CP is not a clinically relevant perpetrator for OATP1B1 and OATP1B3. The primary mechanism of RSV–CP DDI can be attributed to the inhibition of intestinal BCRP by CP combined with the inhibition of hepatic BCRP by CPC (Figure 7). The latter is likely to be more clinically relevant and be a contributing factor for increased hepatotoxicity in the presence of DDI.

## Data Availability

The raw data supporting the conclusions of this article will be made available by the authors, without undue reservation.
