# Supplementary material for: Evaluation of a Clinically Relevant Drug–Drug Interaction Between Rosuvastatin and Clopidogrel and the Risk of Hepatotoxicity
Source: Front Pharmacol. 2021 Sep 27;12:715577. doi: 10.3389/fphar.2021.715577 (PMC8504577; doi:10.3389/fphar.2021.715577)
Supplement: Supplementary file 1 [file DataSheet1.DOCX]

Supplementary Material

**Supplementary Table 1.** Validation results for accuracy, precision and dilution integrity in rat plasma (n=6).

| Analyte | Spiked concentration (ng/mL) | Measured concentration | | | |
| --- | --- | --- | --- | --- | --- |
|  |  | Mean concentration± S.D.  (ng/mL) | | Accuracy (bias, %) | Precision  (CV, %) |
| RSV | 4 | 3.70 ± 0.16 | | -7.50 | 4.41 |
|  | 160 | 171.60 ± 3.78 | | 7.25 | 2.20 |
|  | 4000 | 3910.00 ± 99.40 | | -2.25 | 2.54 |
| CP | 0.08 | 0.08 ± 0.01 | | -4.83 | 8.04 |
|  | 0.8 | 0.72 ± 0.03 | | -10.34 | 3.64 |
|  | 8 | 7.82 ± 0.24 | | -2.25 | 3.02 |
| CPC | 4 | 4.00 ± 0.29 | | 0.00 | 7.32 |
|  | 160 | 152.80 ± 5.07 | | -4.50 | 3.32 |
|  | 4000 | 4086.67 ±129.25 | | 2.17 | 3.16 |
| Analyte | Spiked concentration (ng/mL) | Dilution factor | Measured and back-calculated concentration | | |
|  |  |  | Mean concentration ± S.D. (ng/mL) | Accuracy (bias, %) | Precision  (CV, %) |
| RSV | 50000 | 1:20 | 47433.33 ±1504.22 | -5.13 | 3.17 |
| CP | 100 | 1:20 | 95.53 ± 4.62 | -4.47 | 4.84 |
| CPC | 50000 | 1:20 | 51400.00 ±1734.36 | 2.80 | 3.37 |

**Supplementary Table 2.** Pharmacokinetic parameters of hepatobiliary transport study in rats. (n= 3) RSV CL_uptake,liver_ and CL_int,bile_ were calculated by integration plot methods.^[1, 2]^

|  | without DDI | with DDI | (with DDI/without DDI)% |
| --- | --- | --- | --- |
| Plasma C_max_ (ng/mL) | 62133.33 ± 24249.61 | 56033.33 ± 9154.42 | 90.18 |
| Plasma T_max_ (min) | 0.31 ± 0.096 | 0.36 ± 0.096 | 116.13 |
| Plasma AUC_0-5min_ (min*ng/mL) | 71284.17 ± 13097.20 | 102459.72 ± 18047.77 | 143.73 |
| Liver C_max_ (ng/g) | 38866.67 ± 3503.67 | 36840.00 ± 1304.15 | 94.79 |
| Liver T_max_ (min) | 2.00 ± 0.50 | 1.72 ± 0.95 | 86.00 |
| Liver AUC_0-5min_ (min*ng/g) | 122384.16 ± 13075.60 | 141196.71 ± 13631.53 | 115.37 |
| Liver AUC_0-5min_/Plasma AUC_0-5min_ (mL/g) | 1.72 ± 0.18 | 1.38 ± 0.13 | 80.23 |
| Bile_0-5min_ (μg) | 257.57 ±104.42 | 218.80 ± 138.59 | 84.95 |
| Bile_0-5min_/Liver AUC_0-5min_ (g/min) | 2.10 ± 0.85 | 1.55 ± 0.98 | 73.81 |
| CL_uptake,liver_ (mL/min/g) | 0.84 ± 0.45 | 0.40 ± 0.15 | 47.62 |
| CL_int,bile_ (g/min) | 2.96 ± 1.50 | 1.80 ± 1.10 | 60.81 |

#
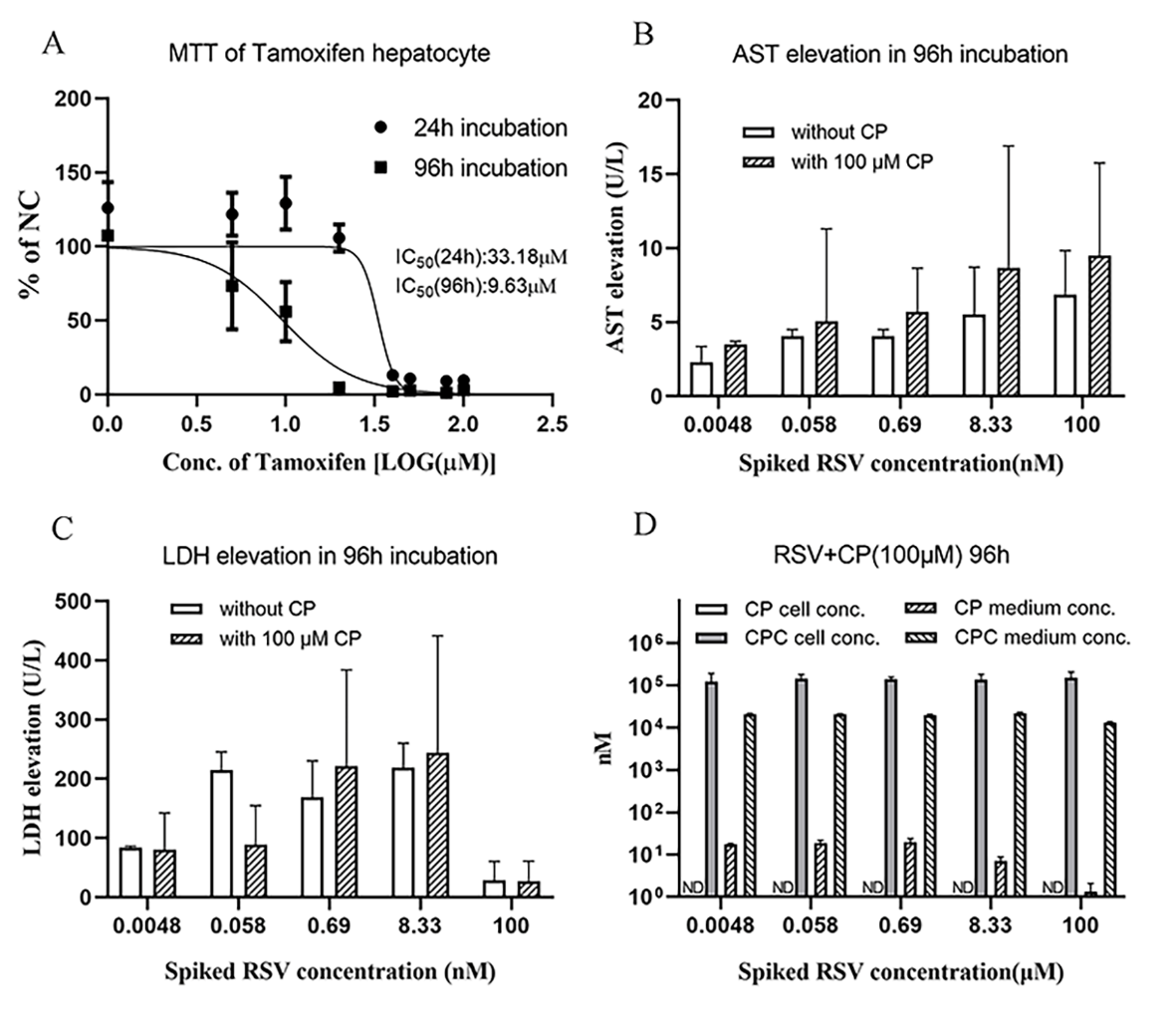


**Supplementary Figure 1.** Effects of tamoxifen on the viability of human primary hepatocytes **(A)**. AST **(B)** and LDH **(C)** levels in human primary hepatocytes after 96 h incubation in the presence and absence of CP. CP and CPC concentration in hepatocytes in the presence of RSV and 100 μM CP **(D)**.

**REFERENCE:**

1. Shingaki, T., Takashima, T., Ijuin, R., Zhang, X., Onoue, T., Katayama, Y., et al. (2013). Evaluation of Oatp and Mrp2 activities in hepatobiliary excretion using newly developed positron emission tomography tracer [11C]dehydropravastatin in rats. *J Pharmacol Exp Ther.* 347, 193-202. doi: 10.1124/jpet.113.206425.

2. Nezasa, K., Higaki, K., Matsumura, T., Inazawa, K., Hasegawa, H., Nakano, M., et al. (2002). Liver-specific distribution of rosuvastatin in rats: comparison with pravastatin and simvastatin. *Drug Metab Dispos.* 30, 1158-63. doi: 10.1124/dmd.30.11.1158.
